# Parental Perception of Vocal Contact with Preterm Infants: Communicative Musicality in the Neonatal Intensive Care Unit

**DOI:** 10.3390/children8060513

**Published:** 2021-06-17

**Authors:** Maria Grazia Monaci, Maya Gratier, Colwyn Trevarthen, Didier Grandjean, Pierre Kuhn, Manuela Filippa

**Affiliations:** 1 Department of Social and Human Sciences, University of Valle d’Aosta, 11100 Aosta, Italy; m.monaci@univda.it; 2 Laboratoire Ethologie Cognition Développement, UPL, Université Paris Nanterre, 92000 Nanterre, France; m.gratier@gmail.com; 3 School of Psychology, University of Edinburgh, Edinburgh EH8 9YL, UK; c.trevarthen@ed.ac.uk; 4 Swiss Center for Affective Sciences, Neuroscience of Emotion and Affective Dynamics, Department of Psychology, Faculty of Psychology and Educational Sciences, University of Geneva, 1205 Geneva, Switzerland; didier.grandjean@unige.ch; 5 Centre Hospitalier, Service de Médecine et Réanimation Néonatale, Hôpital de Hautepierre, Universitaire de Strasbourg, 67081 Strasbourg, France; pierre.kuhn@chru-strasbourg.fr

**Keywords:** preterm infants, maternal voice, early intervention

## Abstract

In this study, we evaluate mothers’ subjective experience of speaking and singing to their infants while they are in their incubators. We also discuss the relevance of the theoretical framework of Communicative Musicality for identifying the underlying mechanisms that may help explain its beneficial effects, both for parents and infants. Nineteen mothers talked and sung to their stable preterm infants in the incubators, for 5 min each, in three sessions over a period of 6 days. After each session, mothers were asked to assess in a self-report questionnaire the ease and the effectiveness of addressing their infants by speaking and singing and their prior musical experience. Perceived ease and effectiveness in communication were found to increase progressively from one session to the next. Mothers rated the speech to be increasingly more effective. This intuitive mean of interaction between parents and infants could be encouraged and supported by the nurses and the medical staff. Furthermore, individual musical experience affects perceived ease of communicating vocally with infants after a premature birth and should thus be encouraged during pregnancy.

## 1. Introduction

Preterm birth entails a disruption of contact between mothers and preterm infants in the newborn intensive care unit (NICU) environment, with potential long-term effects on preterm infants’ neurodevelopment [1]. Moreover, the vast majority of the NICU environment exposes preterm infants to excessive deleterious sensory stimuli, such as frequent loud and high-pitched sounds, and deprives them of naturally meaningful sensory stimuli, such as the mother’s voice. Noise in the NICU triggers stress responses, reduces physiological well-being, and disrupts sleep [2,3].

In the last decades, single and family rooms mitigated the noise exposure in the NICUs [4,5], but the reduction of noise is still a challenge.

Within this complex ecological environment, emotional and multisensory exchange between preterm infants and their parents becomes essential for the infant’s developing brain [1,6]. A number of early intervention programs actively involving families in early contact with and care of their preterm infants have been shown to have both short- and long-term beneficial effects [7]. They support social interaction between parents and infants, and they aim at sustaining natural physiological and behavioral regulatory processes in the dyad, favoring the formation of attachment relationships that are essential for optimal development [8].

Early vocal contact, involving the communication of affectionate feelings through the privileged medium of the voice, whether through songs or speech, could constitute a non-invasive strategy to moderate the deleterious effects of a lengthy hospitalization, and may have effects akin to other forms of early contact on vitality—better heart rate variability, increase in calm awake states and eye opening [9]. Early vocal contact in the NICU has been shown to carry benefits on preterm infants (for a review see [10], increasing their autonomic stability [11], and on mothers, decreasing stress, anxiety and increasing emotion in maternal voice [12,13,14]. In the long-term, the early exposure to meaningful voices before term-equivalent age is associated with better cognitive, language and communicative development during toddlerhood [15]. However, the specific mechanisms underlying these beneficial effects have not yet been clearly identified.

Maternal subjective experience of using their voice—singing and speaking—has been investigated and discussed in previous qualitative studies [16,17,18,19,20].

In this paper, we explore the way mothers perceive the experience of vocally addressing their infant during hospitalisation in the NICU through direct speaking and singing. We propose that the mutual benefits for mothers and infants of such a vocal contact are rooted in the shared ability to move with an innate ‘Communicative Musicality’ [21]. This theory posits that, when in close contact, human infants can grasp the emotions and expressiveness of caring adults and can express their own states of social interest to them by regulating and sensing the vitality dynamics of body movements [22].

### Communicative Musicality in the NICU: The Theoretical Framework

Newborns are equipped by their development in utero to perceive some fundamental aspects of the world they are born into. Their sensory systems become active long before birth [23]. After birth, they are also highly motivated to seek out the attention and affective engagement of social partners [24]. Naturalistic observation of infants with caring adults directing affectionate attention to them—through sensitive actions of gaze, touch or bodily handling—has shown that from the earliest moments of life out of the womb, infants are active partners, listening intently and expressing interest in others with knit-brow expressions and “pre-speech” mouth movements [25], calling and responding with vocalizations that rapidly acquire resonance and well-formed intonation contours and smiling and adjusting their posture in a coordinated process of matching emotion [26,27,28]. Newborns are endowed with special competencies adapted for social engagement and the formation of loving attachments: they may imitate expressions of another person’s intention and feelings signaled by movements of the head, face, mouth, eyes and hands, clearly discriminating different body movements and their motivation [29,30]. Parents’ affectionate speech is ‘musical’ in ways adapted to fit the babies’ behaviors who, in turn, respond with subtly timed vocalizations [31]. From birth, newborns prefer the higher pitch range of the female voice and its affectionate modulations [32]. Mechthild and Hanuš Papoušek described the mother’s ‘intuitive parenting’ speech as having ‘musicality’ [33]. This framework is at the base of several music therapy interventions for preterm infants and their parents in the NICU [34], positing that the use of music, parental singing and aspects of a healthy relationship can promote optimal infant development, facilitate secure attachment with primary caregivers and decrease maternal anxiety [35].

Preterm infants in the NICU are responsive to their sensory and social environment. They are sensitive to infant-directed speech, which helps them to achieve a quiet, attentive state and which leads to increased eye opening [11,36,37]. At about 35 weeks’ gestational age, preterm infants prefer and are more engaged by maternal infant-directed speech than to adult-directed speech [38].

The main purpose of the present study is to investigate the way mothers perceive the ease and efficacy of their own speaking and singing to their preterm infants in the NICU and how it evolves over repeated experiences. After preterm delivery, mothers often experience negative feelings, and their physical and emotional conditions may be unstable.

However, the repeated and constant parental participation to early intervention programs, in particular skin-to-skin contact, showed beneficial effects both in infants and in parents. Fathers and mothers acquire new parental skills and decrease their stress levels [39,40,41].

Investigating the way that parents experience and perceive an early family-based intervention, especially with very young infants during periods of hospitalization, is a complementary assessment of its effectiveness in supporting the baby’s development. We hypothesized that mothers would find it both easier and more useful to speak and sing to their infant as a function of repeated sessions. Finally, it has been shown that parents with prior formal or informal musical experience are more likely to sing to their infants [42]. Thus, we hypothesized that mothers’ prior musical experience would affect the value they attributed to the experience of singing.

## 2. Materials and Methods

### 2.1. Participants

The study was a part of a larger study aiming at evaluating the effects of the early vocal contact on preterm infant’s physiological stability [11]. It was conducted in a level II NICU at the Parini Hospital (Aosta, Italy), limited to newborn infants who were more than 29 weeks’ gestational age and/or weighed more than 1000 g at birth. For participation, a stable medical condition was required for preterm infants (absence of mechanical ventilation, additional oxygen and specific pathological conditions). An absence of mental disease and depressive symptoms was required for mothers. Mothers with a history of substance abuse were excluded. A detailed report on the demographics of the sample can be found in Filippa et al., 2013 [11]. The sample included 9 boys and 10 girls (34.4 ± 4.3 (32–38) at test). Nineteen mothers were included in the study, but the number of participants at each session decreased from the first session (19, 10 female), to the second (18, 10 female) and the third (13, 7 female). Most of the mothers in the sample were Italian nationals and spoke Italian (16/19) and had stable employment (17/19). In all the families, the fathers were present, but they did not take part in the study. The mothers’ mean age was 31.5 ± 3 years at the time of the infants’ birth. The official Hospital Ethical Committee reviewed and approved the study (I.C. n. 12453), and informed consent was obtained from both parents.

### 2.2. Procedure

Mothers were asked both to speak and to sing to their infants continuously for 5 min in each of the two modes (10 min in total). They were free to choose to begin with speaking or with singing and the order was inverted in the following session. The intervention occurred every day at the same hour, between 1:00 and 2:00 p.m., more than one hour after the first afternoon feed. As different measures were collected 10 min before and 10 min after the vocal contact, we decided to limit the intervention to 10 min for feasibility reasons.

As the aim of the larger study was to evaluate the effect of intuitive parental vocal communication on preterm infants [11], no specific support was given to mothers. The instructions were very simple. Mothers were asked to open the incubator’s windows, to vocally address to the infants in the incubators, and to refrain from touching them.

Mothers could choose the contents of the speech and songs addressed to their infant, and they were encouraged to speak/sing in their native language. The researcher was present only if expressly requested by the mothers.

### 2.3. Methods

Mothers were asked to fill a self-report questionnaire, lasting about 15 min, after each Vocal Contact session. They were asked to complete the questionnaires separately for speaking and singing, so that each mode of communication was individually evaluated. They had to rate the ease of speaking/singing on three items (How difficult was it to speak/sing to your preterm infant? Did you feel comfortable while speaking/singing to your infant? Did you feel like you were close to your infant while you spoke/sang to him/her? Each scored on a ten-point scale from 1 ‘not at all’ to 10 ‘very much’) in each of the three repeated sessions. Scores of the three items were averaged into a single composite variable for Ease of speaking (Cronbach α = 0.77) and singing (Cronbach α = 0.76).

Secondly, mothers had to rate the effectiveness of speaking/singing, which was assessed with three items (Did you feel that there was a kind of communication while speaking to your infant? Did you feel that your infant was listening while you were speaking/singing? Did she/he answer while you spoke/sung to her/him? Each item had four possible responses: Yes, No, perhaps, I don’t know). The Yes answers were scored 1, the Perhaps answers were scored 0.5, the No and I don’t know answers were scored 0, and a composite score was obtained by summing the scores (Cronbach α = 0.78 for speech and α = 0.86 for singing, respectively).

Finally, maternal musical experience was assessed using six items, four items with dichotomous answers and two items with a 10-point Likert-type scale (Is music present in your daily life? If yes, how much? Do you like singing? If yes, do you think that singing is a way to communicate emotions? Did anyone sing for you when you were a baby? If yes, was it a pleasant experience? see Table 1). Summing the Yes answers to the four dichotomous items and averaging the variables, a global index of maternal experience (α = 0.67) was used in the analyses.

## 3. Results

### 3.1. Ease of Singing and Speaking

**Figure 1:**
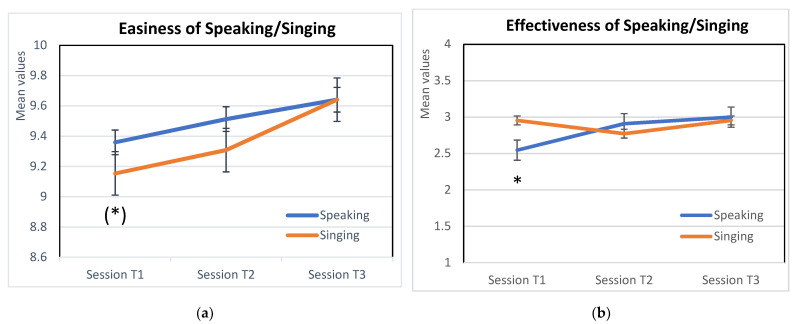
Mean values of (**a**) Perceived ease and (**b**) Perceived effectiveness of maternal speaking and singing to preterm infants in the NICU across the three sessions. * *p* < 0.05; (*) tendentially significant, *p* = 0.65.

An ANOVA for repeated measures with two (Mode: Singing/Speaking) x three (Sessions: T1/T2/T3) within factors compared the modality of communication and the three sessions. Only participants with all three sessions were included in the analyses. As shown in Figure 1, the mean values of ease of singing and speaking significantly increased across the three sessions (the within factor Time is significant: F_(12, 1)_ = 5.59, *p* < 0.05, h^2^ = 0.39). No significant differences emerged between the two conditions and no significant interaction was observed. The initial tendentially significant difference of ease between Speaking and Singing (t = 1.96, *p* = 0.065), in the direction of a perception of greater ease for speaking, disappeared in the last two sessions (see Figure 1).

### 3.2. Effectiveness of Singing and Speaking

The same ANOVA for repeated measures was performed on the effectiveness measures. The perceived effectiveness of communication through Singing/Speaking to the preterm infant showed no significant effect of the within factors Time and Type; however, a tendentially significant interaction effect was observed (F_(1a,1)_ = 3.25, *p* = 0.058). As reported in Figure 1b, at the first session, Speaking was perceived as less effective than Singing. During the following repetitions, the perceived effectiveness of Speaking rapidly increased, and in the last two sessions, mothers perceived similar Effectiveness for the two forms of vocal communication. A post-hoc comparison with paired t-tests revealed that the difference in perception between Singing and Speaking was significant only at Session1 (t_(18)_ = 2.12, *p* < 0.05).

### 3.3. Influence of the Maternal Musical Experience on the Perception of Ease and Effectiveness

Maternal Musical Experience is reported in Table 1. As reported in Table 2, it significantly correlated with Ease of Speaking, increasingly across the sessions, while the correlation with Ease of singing is moderate at Session 1 and Session 3, without reaching significance. No significant correlations emerged with the Effectiveness of speaking and singing, at any session.

## 4. Discussion

The aim of this study was to evaluate changes in mothers’ attitudes towards infant-directed vocal expression as they were asked to engage with their hospitalized preterm infants placed in incubators. Parents often cannot immediately hold their infants in close body contact because of medical and other constraints, i.e., specific emergencies due to infections in the NICUs. Singing and speaking to preterm infants may thus help rekindle intuitive modes of communication within the unnatural ecology of the NICU.

This study shows that mothers, if encouraged to talk and sing for their preterm infants, gradually gain confidence in their capacity to communicate vocally with their stable preterm infants over a period of one week. It appears that the more they engage vocally with their infants, the easier it feels.

Maternal singing was initially perceived as more effective for communicating than speech. This finding supports the idea that maternal singing is conceived by parents as an effective mean of soothing and regulating infants’ emotions [43]. However, this difference gradually disappears with repeated experience, by an increase of the perceived effectiveness of infant-directed speech. This increase may reflect the functional flexibility of speech with different qualities of intonation in response to preterm infants’ behavioral cues.

Although infant-directed speech and singing seem to be equally effective for capturing infant attention [44,45], they differ in their efficacy for sustaining attention and regulating emotion [46]. In a previous study comparing maternal speaking and singing directed to preterm infants [11], the authors showed that infants’ arousal was increased by maternal speech, but not by maternal singing. This finding may be explained by the fact that, when singing in the NICU, mothers reproduce the musical features of lullabies, which are known to be more effective than maternal speech in lowering arousal in infants following a stressor [47].

Finally, we found that maternal informal musical experience influences the perceived ease in communicating vocally with the infant, especially in the speaking condition. This result, relating informal musical experience with speaking and not only with singing, suggests that speech and songs directed to infants are deeply linked. More specifically, an informal musical experience can have effects in the general practice of vocal communication, not specifically for singing. This finding is supported by several instances of research evaluating the impact of a musical environment on the development of musical parental competences during infant’s development [48]. Moreover, it is suggested that parental ability to communicate verbally and vocally with their infant can also be encouraged indirectly through prenatal musical experience, such as singing in groups and acquiring a repertoire of songs and musical play. Musical experience during pregnancy, for both mothers and fathers, can help parents to address their infants in meaningful and affectionate ways, and may constitute a source of resilient competence to be used in complex contexts such infant illness, hospitalization or prematurity [49].

A major limitation of the present study is its small sample size at the third session of the vocal contact intervention. Moreover, we only included stable preterm infants. The restrictions imposed on the population included in this study could bias perception of ease and efficacy of early vocal contact.

Finally, not all the mothers accepted to participate to the study, and the protocol acceptability could reasonably constitute an aspect of attrition for measuring the maternal perception of Early Vocal Contact. However, the present study evaluated the maternal perception in its dynamical changes over the three sessions.

Another limitation is due to the fact that we did not evaluated the potential interference between the changes in the baby’s state over the three sessions and the maternal evaluation of the effectiveness for communicating.

In future studies, parents with depressive symptoms and in socially vulnerable conditions should be included, in order to specifically assess the impact of vocal contact in adverse conditions [21]. Fathers were excluded in this exploratory study, as their presence in the NICU was limited during the day. However, the paternal voice modulates on preterm infant’s behaviors, with specific acoustic characteristics when compared to mothers [50], suggesting the need of a complementary paternal intervention in the NICU [41]. Further research is needed for developing scales, questionnaires and qualitative methods for investigating parental perception of early family-based interventions. Finally, further research in different cultural contexts is encouraged to evaluate the effect of culture on maternal musical experience and on her ability to use singing as an effective communication tool.

## Figures and Tables

**Table 1:** Maternal musical experience.

	N (Yes Answers/Total)	(%)
Is music present in your daily life? (a)	18/19	94.7%
If yes, how much? (b)	7.05(2.04)	
Do you like singing? (a)	14/19	73.7%
If yes, do you think that singing is a way to communicate emotions (b)	9.37(1.30)	
Did anyone sing for you when you were a child? (a)	14/19	73.7%
If yes, was it a pleasant experience (a)	12/19	63.2%

(a) Yes/no, yes answers and percentage reported; (b) 10-point Likert-type scale, mean and (standard deviation) reported.

**Table 2:** Pearson correlation between ease and efficacy of singing/speaking and maternal musical experience in the three sessions.

	Session T1 (*n* = 19)	Session T2 (*n* = 18)	Session T3 (*n* = 13)
Ease of speaking	0.46	0.63	0.77
Ease of singing	0.35	0.10	0.29
Efficacy of speaking	−0.30	−0.26	.^a^
Efficacy of singing	0.04	−0.24	−0.27

Note. ^a^ All the 13 mothers at the Session 3 answered Yes. In bold significant correlations at *p* < 0.05.

## Data Availability

Data will be available on request.
